# Use of Eye-Tracking Technology by Medical Students Taking the Objective Structured Clinical Examination: Descriptive Study

**DOI:** 10.2196/17719

**Published:** 2020-08-21

**Authors:** M D Grima-Murcia, Francisco Sanchez-Ferrer, Jose Manuel Ramos-Rincón, Eduardo Fernández

**Affiliations:** 1 Facultad de Medicina University Miguel Hernández Elche Spain

**Keywords:** visual perception, medical education, eye tracking, objective structured clinical examination, medical evaluation

## Abstract

**Background:**

The objective structured clinical examination (OSCE) is a test used throughout Spain to evaluate the clinical competencies, decision making, problem solving, and other skills of sixth-year medical students.

**Objective:**

The main goal of this study is to explore the possible applications and utility of portable eye-tracking systems in the setting of the OSCE, particularly questions associated with attention and engagement.

**Methods:**

We used a portable Tobii Glasses 2 eye tracker, which allows real-time monitoring of where the students were looking and records the voice and ambient sounds. We then performed a qualitative and a quantitative analysis of the fields of vision and gaze points attracting attention as well as the visual itinerary.

**Results:**

Eye-tracking technology was used in the OSCE with no major issues. This portable system was of the greatest value in the patient simulators and mannequin stations, where interaction with the simulated patient or areas of interest in the mannequin can be quantified. This technology proved useful to better identify the areas of interest in the medical images provided.

**Conclusions:**

Portable eye trackers offer the opportunity to improve the objective evaluation of candidates and the self-evaluation of the stations used as well as medical simulations by examiners. We suggest that this technology has enough resolution to identify where a student is looking at and could be useful for developing new approaches for evaluating specific aspects of clinical competencies.

## Introduction

The objective structured clinical examination (OSCE) is an evaluation of medical students that aims to assess candidates’ skills and attitudes in certain clinical situations. This goal is different from that of typical written exams, which primarily evaluate knowledge. Basically, evaluating clinical competencies entails objective measurement of whether a candidate has correctly used and applied theoretical knowledge [1]. The exam is used not only for undergraduate students but also in the graduate context [2,3] and in different medical specialties [4,5]. These tests have been in use since the 1970s and have proven to be a reliable and valid tool for evaluation, even when using the same stations [6]. The OSCE is particularly important for evaluating and teaching semiology skills and training the clinical eye of students [7].

The OSCE incorporates diverse evaluation instruments, which are arranged in successive stations that simulate real clinical situations [8]. The strength of this format lies in the mixed-methods evaluation, which allows examiners to explore three of the four levels of Miller’s pyramid: to know, to know how, and to show how [3]. The number of stations ranges from 5 to 20, according to the objectives being evaluated [9].

In Spain, various medical schools have not implemented this testing method uniformly, with universities in Catalonia—as with many medical schools worldwide [10]—gaining the most experience in the more than 20 years since they began using the OSCE [11].

Recently, the National Deans Conference in Spain approved a 20-station OSCE that all sixth-year medical students must take. The general characteristics of the test are practical character; oriented toward evaluating the professional competencies of the candidate relative to the specific competencies of the medical degree, established by the Order ECI/332/2008 (published in the Official State Gazette on February 15, 2008); performed through the resolution of clinical cases; and having the objective of demonstrating clinical skills.

The OSCE explores diverse areas of evaluation through a range of methodologies. The stations include simulated and standardized patients, mannequins, short-answer questions, performance of complementary examinations adjusted to the particular case, clinical report writing, structured oral examinations (SOEs), skills and procedures, computer functions, and simulators. The OSCE entails a considerable collective effort both for candidates and examiners; however, several studies have also reported good cost-effectiveness [12] compared to traditional testing [9].

Video recording for clinical training purposes is already used successfully, including to evaluate procedures, techniques, or diagnostic tests [13,14]. However, the technique proposed in this study is different from these recordings and has substantial implications for teachers [15]. Using an eye tracker, we can view and analyze candidates through their own unique perspective, similarly to how this technology is used for training in gastroscopy or locoregional anesthesia, alone or in conjunction with conventional video [16], or in the field of radiology [17,18]. Consequently, this device makes it possible to perform a qualitative analysis of the test (also allowing examiners to self-evaluate their station) and to objectively analyze those elements that, due to the test design, may introduce subjectivity in the examination, such as the relationship with different speakers or the dynamics of evaluating complementary medical tests [19].

With this in mind, we designed a feasibility study to assess the real possibilities of eye tracking as a tool for teaching and evaluation in the OSCE, according to each station model. To the best of our knowledge, this is the first study to use eye-tracking glasses in each of the 20 OSCE stations. Our main goal is to identify whether this technology can be used in an examination as extensive as the OSCE. In addition, we analyzed the opportunities for evaluating students according to the type of station, the usefulness perceived by the teachers, and the teaching opportunities for students. The hypothesis was that this technology is useful in teaching, specifically in the OSCE.

## Methods

This is a descriptive study on the use of eye-tracking glasses during the OSCE. We carried out an OSCE of 117 sixth-year medical students at the Medical School of Miguel Hernández University Spain in June 2017. The OSCE consisted of a circuit of 20 stations or situations, and the candidates had to move consecutively through all of them, spending 9 minutes on each, with 2-minute rest periods between each new station. The stations and the skill areas included were history taking, physical examination, doctor-patient communication, clinical report writing, clinical judgement, technical skills, preventive activities, and ethical-legal issues (Table 1). The students had completed a shorter OSCE in the third year.

The exam was carried out in parallel rounds of 23 candidates each. All rounds were conducted on the same day. Two rounds were held in the morning and three rounds in the afternoon (one with 25 students). Forty consultation areas were prepared in line with the needs of the specific stations, each equipped with a computer program created for candidate evaluation. The simulated patient or examiner completed a checklist of evaluation items for the skill area being assessed. Exams were continuous, with rotations communicated through a computer program with speakers. The exams lasted 4.5 hours. Candidates were not permitted to have any electronic items with them (including mobile phones or watches), and all had a white coat, stethoscope, pencil or pen, and two blank sheets of paper.

**Figure 1:**
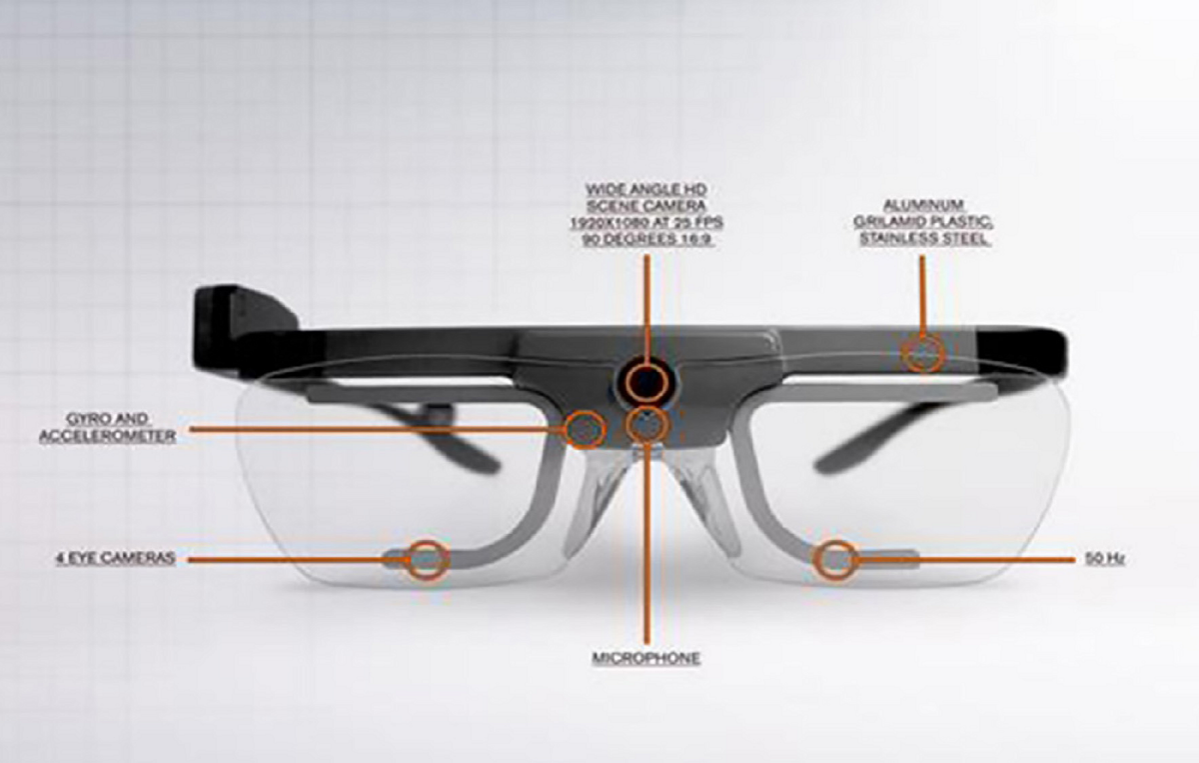
Portable eye tracker used.

The portable eye trackers used were the Tobii Glasses 2 [13,16] (Figure 1). The system was calibrated upon start up for each wearer. This device weighs 45 g and has a 160° field of vision, with minimal vision loss with extreme eye movements. Both image and sound were recorded simultaneously. The recordings were saved on memory cards for subsequent analysis and transmitted through a wireless network to a projector or computer for real-time visualization by the teachers (recording delay of 1 second via Tobii Glasses Controller). Some of the research questions were can users recognize the purpose of each specific station; if not, what are the obstacles; what was the main focus of attention in each station; were the medical images provided easy to read and understand?

The videos for each station were edited to eliminate the waiting periods and to include only the time period from which the participants read the case being evaluated prior to entering the consultation area. The audio recordings at each station were also homogenized so that the volume levels were approximately the same for all stations.

**Table 1:** Station, type of station, and skills map for the objective structured clinical examination.

Station number	Station	Type	HT^a^	CE^b^	TSP^c^	CS^d^	CJ^e^	DP^f^	IR^g^	EL^h^	Total
1	MSP^i^ of the digestive tract	SP^j^	65.5	24.1	0.0	10.3	0.0	0.0	0.0	0.0	100
2	MSP of the digestive tract	Report	0.0	0.0	0.0	0.0	34.8	65.3	0.0	0.0	100
3	Infectious disease pathology	SOE^k^	0.0	0.0	0.0	0.0	0.0	0.0	90.0	10.0	100
4	Emergency medicine	M-P^l^	0.0	35.0	55.0	0.0	0.0	0.0	0.0	10.0	100
5	Legal medicine	Report	0.0	0.0	0.0	0.0	0.0	38.1	61.9	0.0	100
6	Emergency	SP	0.0	96.8	0.0	3.2	0.0	0.0	0.0	0.0	100
7	MSP of the nephrourinary system	M-P	0.0	0.0	55.0	0.0	35.0	0.0	0.0	10.0	100
8	Psychiatry	SP	22.6	35.5	0.0	12.9	29.0	0.0	0.0	0.0	100
9	MSP of the musculoskeletal system	M-P	0.0	57.2	28.6	0.0	14.3	0.0	0.0	0.0	100
10	Pediatrics	SOE	0.0	0.0	0.0	0.0	42.1	0.0	47.4	10.5	100
11	Infectious disease pathology	SP	43.3	13.3	0.0	10	33.4	0.0	0.0	0.0	100
12	MSP of the endocrine system	SOE	20	0	30	0	10	0	40	0	100
13	Surgery	M-P	0.0	0.0	83.3	0.0	0.0	0.0	12.5	4.2	100
14	Gynecology and obstetrics	SP	50.0	0.0	0.0	15.0	25	0.0	10	0.0	100
15	Gynecology and obstetrics	M-P	0.0	15.8	26.3	0.0	47.4	0.0	10.5	0.0	100
16	Pediatrics	SP	38.5	30.8	0.0	11.5	11.5	0.0	7.7	0.0	100
17	MSP of the respiratory system	Report	0	0	0	0	70	30	0	0	100
18	MSP of the cardiovascular system	SP	44	36	0	12	8	0	0	0	100
19	MSP of the cardiovascular system	Report	0	0	0	0	100	0	0	0	100
20	Microbiology and legal medicine	Report	0.0	0.0	0.0	0.0	38.9	0.0	22.2	38.9	100

^a^HT: history taking.

^b^CE: clinical examination.

^c^TSP: technical skills and procedures.

^d^CS: communication skills.

^e^CJ: clinical judgement, diagnostic and therapeutic management plan.

^f^DP: disease prevention and health promotion.

^g^IR: interprofessional relationships.

^h^EL: ethical issues—legality and professionalism.

^i^MSP: medical and surgical pathology.

^j^SP: standardized patients.

^k^SOE: structured oral examination.

^l^M-P: mannequin-procedure.

The analysis was performed using Tobii Glasses Analysis Software and included a quantitative study of gaze points and the order that they appeared for each subject. We also used heat maps for visualization of eye tracking. Thus, we identified the areas of interest in each station and the percentage of the total area that they occupied within the image as a whole. Using these percentages, we calculated the participants’ most frequent gaze locations, the number of gazes, and the visual itinerary (ie, the order that the gazes occurred). We also measured pupil dilation, but we found that it was extremely difficult to control luminance and maintain stable lighting conditions in our 20 different experimental conditions. Hence, we discovered that this particular measure was not appropriate for this type of study. Results were exported to Excel (Microsoft Corp) for statistical analysis.

Finally, each of the station examiners (N=30) viewed the videos and completed a questionnaire to assess the usefulness of the eye tracker for each type of station in the visualization and interpretation of complementary tests, the assessment of the attitude of simulated patients and examiners, the evaluation of the candidate, the characterization of empathy and eye contact, the assessment of the design of the physical space where the exam was performed, the stimulation of ideas on training possibilities for examiners, and the external evaluation of the exam. Each of these questions was scored according to the usefulness of the device as follows: 0 not at all useful, 1 a little useful, 2 somewhat useful, and 3 very useful.

The Research Ethics Committee of Miguel Hernandez University approved this study (DMC.JRR.01.17). Verbal consent was obtained from the study participants.

## Results

The OSCE consisted of 7 stations with simulated patients, 5 testing technical skills and procedures with or without mannequins, 5 testing candidates’ abilities to draft a clinical report, and 3 SOEs on practical clinical situations. Of the 20 stations, we obtained useful recordings of 16 (80%). No recordings were made for 3 of the stations because the specific tasks involved writing reports (stations 5, 19, and 20). Furthermore, we encountered technical problems in the recordings associated with station 7 (possibly due to the battery); therefore, we discarded these data.

### Eye Tracking in Stations With Simulated Patients

We evaluated 7 stations with simulated patients (stations 1, 6, 8, 11, 14, 16, and 18). The mean time for history taking was 242 (SD 28.4) seconds. In this group of stations, the eye‑tracking device provided a wealth of usable data, demonstrating the extent to which the candidate *connected* on a visual level with the simulated patient. In this case, the candidate looked mainly at the patient’s eyes and mouth (83.3% of the time).

**Figure 2:**
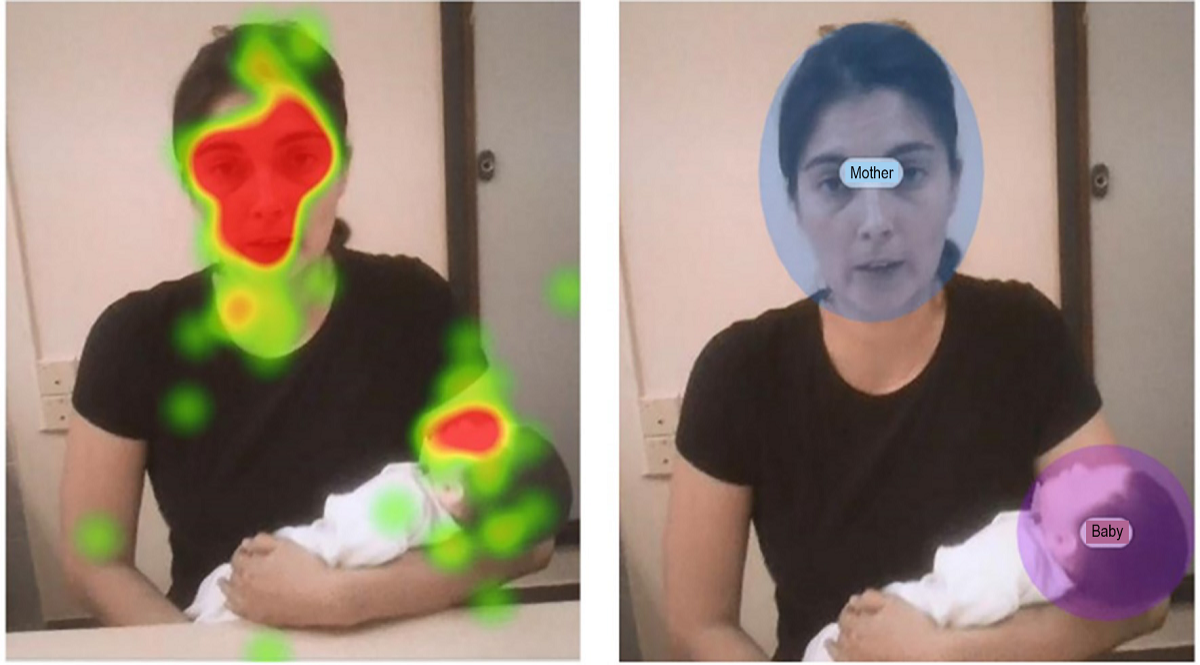
The areas of visual interest were the faces of the mother and the infant.

In station 16, this pattern was of special interest, as it involved a simulated mother with a pediatric mannequin. The areas of visual interest were the faces of the mother and the infant (Figure 2). Of the entire image, the area of the mother’s face was 12.1% of the total, while the infant’s face corresponded to 5.6%. Figure 2 shows a heat map of the areas of interest (in blue and purple) for a given participant. The quantitative result for the time spent looking at both areas was 84.4%: 76.5% for the mother and 7.8% for the mannequin. Thus, of the 220 total gazes, 173 (86.5%) were focused on the mother’s face, 25 (11.3 %) on the infant’s face, and 22 (10.0%) on other locations. Moreover, the length of the gazes on other locations was shorter than for the two main areas of interest.

### Eye Tracking in Technical Skills With or Without Mannequins

We evaluated 5 stations (1, 4, 7, 9, and 15) associated with the assessment of technical skills. Given that the students were constantly moving in these stations, for example, in the station associated with cardiopulmonary resuscitation (CPR) or in the station to evaluate suturing skills, it was difficult to analyze the results. Analysis of these data, therefore, required manual coding of the video to quantify the steadiness of the gaze [19], which was overly complex and outside of this research’s scope. The subjective evaluation allowed us to observe the order used by the student in CPR and how, before each action, the student’s gaze was fixed on the next task. We also identified the elements of the station that were not visualized by the student.

### Eye Tracking in Image-Based Clinical Reports

In drafting clinical reports, for example, on electrocardiograms, x-rays, or other images, the eye tracker was not useful, as it only showed the candidate writing. However, in the stations evaluating a diagnostic test (for example, a chest x-ray), we were able to measure the time the candidates spent evaluating the image and the potential relationships between the focus of attention and the student’s interpretation of the clinical case.

**Figure 3:**
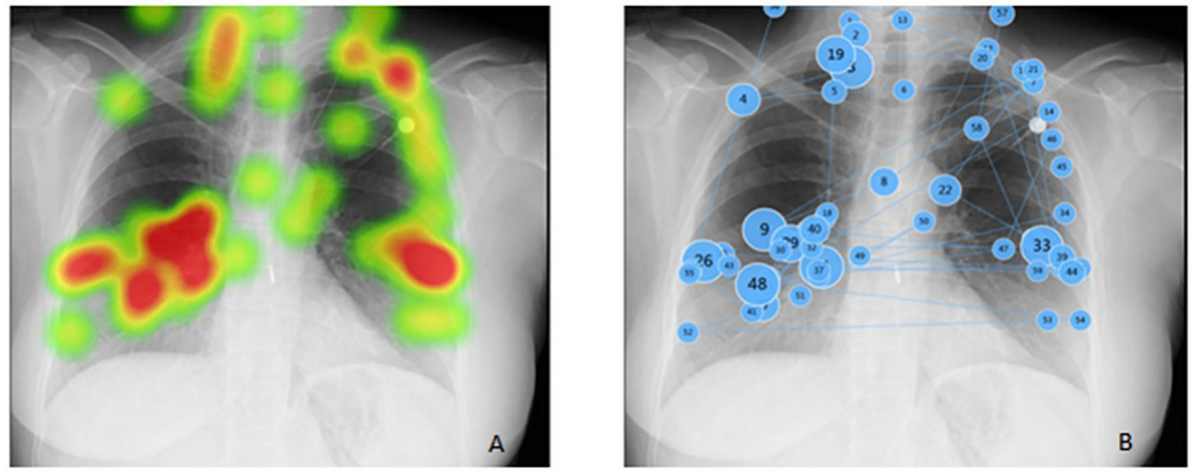
The time spent and location of the gaze by the medical student looking at chest x-ray.

Figure 3 shows a representative example for station 19, dealing with a clinical report based on an image prompt (chest x-ray). The time spent by the medical student looking at the chest x-ray for this particular subject was 27.61 seconds. The area of interest, the hila, comprised 15.9% of the total area of the image, and the student spent 34% (9.5 seconds) of the time on this particular area (the initial gazes focused on the upper left zone). Furthermore, of the 59 total gazes, 26 were on the hila, and these gazes were also the longest.

**Figure 4:**
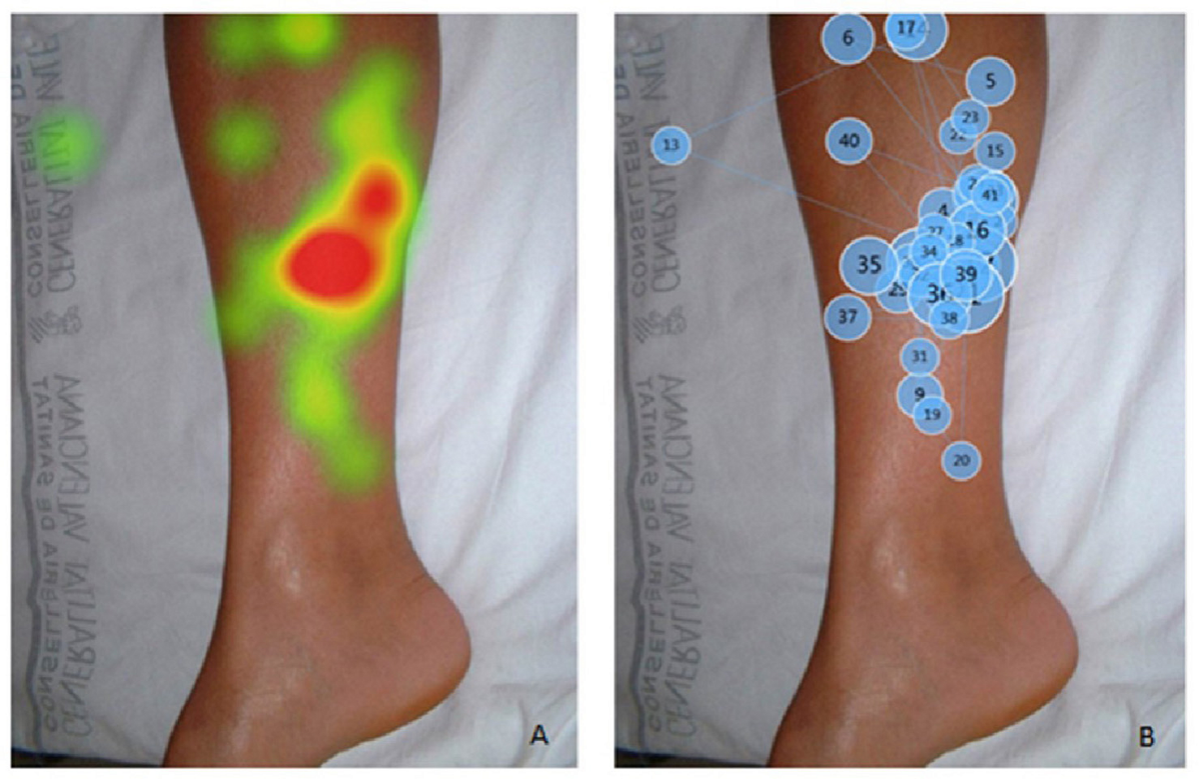
The time spent and location of gaze by the medical student looking at an image of skin lesions on a lower limb.

Figure 4 illustrates a different finding for station 11, which involved a simulated patient and an image of skin lesions on a lower limb. The mean time looking at this image was 17.8 seconds. In this case, we found no apparent areas of interest or systematic approach in the order of the gazes. Eye tracking was also useful for assessing the elements of the station not looked at by the candidates.

### Evaluating the Usefulness of Eye Trackers According to the Station

Table 2 shows the mean subjective perceptions of the examiners regarding the potential value of a portable eye tracker in the evaluation of OSCEs. The usefulness of eye tracking appeared to be virtually nonexistent in all domains for the reports, while it was *somewhat useful* for the SOE, the evaluation of the simulated patient and examiner, and the preparation of the candidates. We did not find it useful for other areas of interest.

**Table 2:** Usefulness of portable eye-tracking devices in the OSCE by type of station for 30 station examiners.

Opportunities for eye tracking	Type of station, mode^a^			
	Standardized patients	Mannequin/procedure	Report	SOE^b^
Evaluation of complementary tests	0	1	2	1
Evaluation of simulated patient/examiner	3	3	0	2
Re-evaluation of candidate	2	3	0	1
Empathy/eye contact	3	1	0	1
Design of OSCE^c^ consultation area	3	3	0	0
Candidate preparation for OSCE	2	3	1	2
External evaluation of OSCE	3	3	0	2

^a^Likert scale: 0 not at all useful, 1 a little useful, 2 somewhat useful, 3 very useful.

^b^SOE: structured oral examination.

^c^OSCE: objective structured clinical examination.

In contrast, the eye tracker was *very useful* for stations with standardized patient and mannequin procedures. This was true in practically every domain (with the exception of the evaluation of complementary tests and in the mannequin procedures to measure empathy or eye contact). Furthermore, portable eye tracking appeared to be of particular interest in the re-evaluation of the candidates and in candidate preparation for the mannequin procedures. In addition, these videos have subsequently been used in teaching to provide future students of the test an insight into OSCE assessments.

## Discussion

Our study shows that portable eye-tracking is an applicable and useful tool in the OSCE. In the standardized patient stations, mannequins or pictograms of the procedure were useful in many aspects. Herein is where teachers see more possibilities for their use. In contrast, in the stations where the students had to write reports, the eye tracking was not particularly useful. In addition, since this was the first time this technology was implemented in a complete OSCE, we found that preparation of batteries sufficient for the entire test is essential (recording of one station was lost due to battery issues).

OSCE testing has now been implemented in all the medical schools in Spain, with some programs dating back more than 20 years [11]. The effectiveness of the OSCE has also been demonstrated in various studies [6,9,12]; although, the exams being evaluated are heterogeneous in terms of design, number of stations, length, appropriateness of the simulated patients, the number of elements for assessment in each station, and the scales used to grade candidates’ behavior or attitudes [6,16]. Students value more practical teaching, which includes the OSCE [20].

The use of portable eye-tracking technology has been introduced in medicine more recently [13,15,16,21-24], and many unanswered questions remain, particularly in the field of medical teaching and evaluation. In this context, research using eye tracking is widespread, but research on the use of eye tracking for assessing student performance is scarce. To our knowledge, this is the first study to be undertaken in this field.

Our preliminary results show a wide range of possibilities for future research. The technology may help evaluators to objectively measure the empathy shown by the candidate in the stations with simulated patients by characterizing the features of the candidate’s gaze (eg, establishment of eye contact or steadiness). Although the OSCE is intended as an objective test, some elements are assessed with a certain degree of subjectivity, such as the scores associated with the candidate’s treatment of patients, nonverbal communication, and conversation and empathy [25,26]. The eye tracker with recording represents an improvement over external video recording in that it enables measurement of the amount of time the candidate maintains eye contact with the patient, providing a novel and objective way to evaluate this item. Furthermore, the video is highly useful to professors, allowing them to evaluate the performance of the simulated patient (adherence to the script) and to obtain a comprehensive vision of the station from the candidate’s perspective. This functionality also allows teaching staff to visualize which elements are used in the station, as some are not seen by the candidates due to the object’s location.

Until now, in the stations assessing candidates’ interpretation of image prompts (electrocardiogram, x-ray, photos, etc), we could not know whether candidates responded correctly to the questions as a result of an adequate systematic approach that included a revision of all relevant points or whether their responses were due to chance or previous knowledge of the answer [27]. However, using the eye tracker and the subsequent analysis, we have a better idea of the approach candidates use to reach their conclusion and the basis of their response in the image provided. In this case, studies on eye trackers have evaluated candidates’ interpretation of an electrocardiogram [24] or other medical images [22,23]. However, further research should bear in mind that gaze does not necessarily have a direct correlation with a complex cognitive process.

The usefulness of eye tracking varied according to the station, whether these dealt with image-based clinical reports, images shown to the candidate, simulated patients (as a method to quantify doctor-patient empathy), or procedures or mannequins in the re-evaluation of the candidate. Thus, in the stations using images, we believe that it may be more practical to use optical tracking by means of a device placed directly on the monitor showing the image. As in other studies [17,18], this would allow a more accurate and straightforward quantitative analysis, in which more participants could take part.

On the other hand, the OSCE is stressful for medical students. To help them to prepare this evaluation, universities have created a study guide [28]. In our case, the videos obtained from the eye-tracking glasses were helpful to prepare the OSCE for future students.

We are fully aware that this is a proof-of-concept study and has important limitations. Furthermore, we were not able to compare the results of several students, as eye tracking is an expensive technology, and we only had a few devices available. Nevertheless, our preliminary results suggest that portable eye-tracking devices offer a number of opportunities in the field of OSCE evaluation, including the design, set up, and self-evaluation of the examiners at each station. Moreover, these devices may also provide insight into methods to improve the evaluation of candidates during the exams, particularly in the stations assessing subjective elements. Nevertheless, further studies are still needed.

## Appendix

Multimedia Appendix 1 Sample video captured during the investigation.

## Abbreviations

CPR: cardiopulmonary resuscitation
OSCE: objective structured clinical examination
SOE: structured oral examination
